# Identification of undecylenic acid as EAG channel inhibitor using surface plasmon resonance-based screen of KCNH channels

**DOI:** 10.1186/s40360-019-0324-8

**Published:** 2019-07-17

**Authors:** Ze-Jun Wang, Purushottam B. Tiwari, Aykut Üren, Tinatin I. Brelidze

**Affiliations:** 1 Department of Pharmacology and Physiology, Georgetown University Medical Center, Washington, DC USA; 2 Department of Oncology, Georgetown University Medical Center, Washington, DC USA

**Keywords:** PAS domain, CNBH domain, Drug screening

## Abstract

**Background:**

KCNH family of potassium channels is responsible for diverse physiological functions ranging from the regulation of neuronal excitability and cardiac contraction to the regulation of cancer progression. KCNH channels contain a Per-Arn-Sim (PAS) domain in their N-terminal and cyclic nucleotide-binding homology (CNBH) domain in their C-terminal regions. These intracellular domains shape the function of KCNH channels and are important targets for drug development.

**Methods:**

Here we describe a surface plasmon resonance (SPR)-based screening method aimed in identifying small molecule binders of PAS and CNBH domains for three KCNH channel subfamilies: ether-à-go-go (EAG), EAG-related gene (ERG), and EAG-like K+ (ELK). The method involves purification of the PAS and CNBH domains, immobilization of the purified domains on the SPR senor chip and screening small molecules in a chemical library for binding to the immobilized domains using changes in the SPR response as a reporter of the binding. The advantages of this method include low quantity of purified PAS and CNBH domains necessary for the implementation of the screen, direct assessment of the small molecule binding to the PAS and CNBH domains and easiness of assessing KCNH subfamily specificity of the small molecule binders.

**Results:**

Using the SPR-based method we screened the Spectrum Collection Library of 2560 compounds against the PAS and CNBH domains of the three KCNH channel subfamilies and identified a pool of small molecules that bind to the PAS or CNBH domains. To further evaluate the effectiveness of the screen we tested the functional effect of one of the identified mEAG PAS domain specific small molecule binders on currents recorded from EAG channels. Undecylenic acid inhibited currents recorded from EAG channels in a concentration-dependent manner with IC50 of ~ 1 μM.

**Conclusion:**

Our results show that the SPR-based method is well suited for identifying small molecule binders of KCNH channels and can facilitate drug discovery for other ion channels as well.

**Electronic supplementary material:**

The online version of this article (10.1186/s40360-019-0324-8) contains supplementary material, which is available to authorized users.

## Background

KCNH family of potassium selective and voltage-dependent ion channels is comprised of ether-à-go-go (EAG), EAG-related gene (ERG), and EAG-like K^+^ (ELK) subfamilies [1]. KCNH channels are abundantly expressed in the brain and heart, where they carry out a multitude of functions. ERG channels are one of the primary potassium channels responsible for the heart repolarization [2–5]. Inhibition of hERG channel function, both due to genetically occurring mutations and as an unintended consequence of prescription medication, causes lengthening of the ventricular action potential and associated cardiac arrhythmias [6, 7]. While EAG channels expression is limited under normal conditions, they are overexpressed in tumor tissue [8–10]. Inhibition of EAG currents by an EAG-specific antibody blockade [11, 12], RNA interference [13] and non-specific channel blockers [14] reduces proliferation of cancer cells. ELK channels are expressed in the brain where they contribute to the regulation of neuronal excitability [15] and knockout of ELK channels causes hippocampal hyperexcitability and epilepsy in mouse [16].

**Fig. 1:**
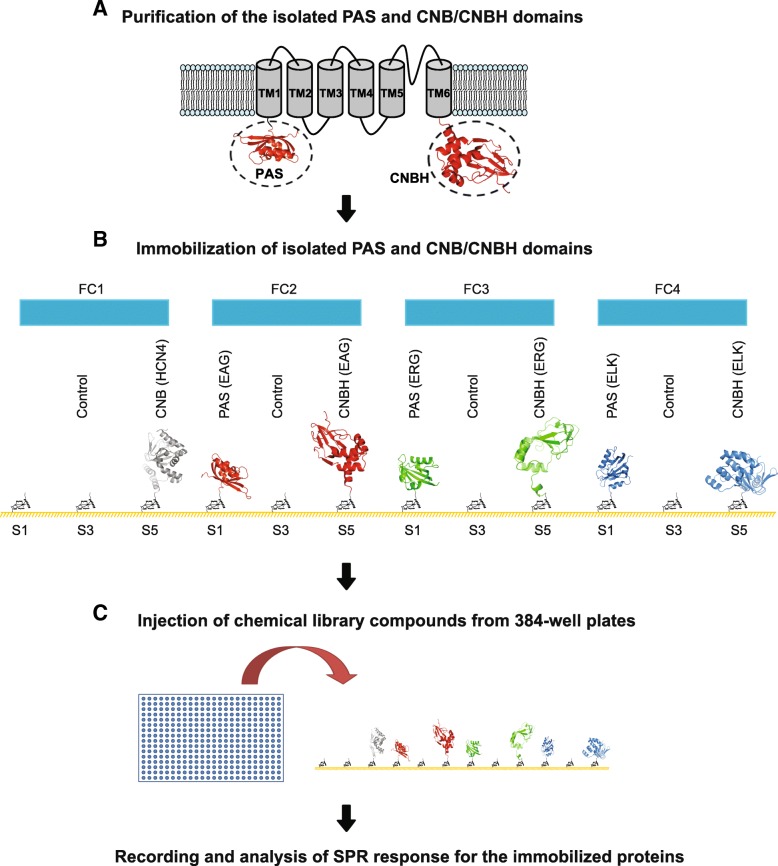
Description of the SPR-based screening method. **a** Isolated PAS and C-linker/CNB(CNBH) domains of HCN and KCNH channels were purified with affinity and size-exclusion chromatography. **b** The purified domains were immobilized on the NTA sensor chip. The NTA chip contained 20 spots for protein immobilization, five spots (S1-S5) per each of the four flow cells (FCs). For each FC, S3 was used as a control spot for non-specific binding, S1 and S2 were used to immobilize the same protein at high density (HD) and low density (LD), respectively, and similarly, S4 and S5 were used to immobilize the same protein at LD density for S4 and HD for S5. Only spots with HD of the immobilized protein are depicted in the figure. For FC1 only spots S4 and S5 had immobilized protein. **c** The Spectrum Library compounds at 50 μM concentration distributed into 384-well plates were injected to the seven immobilized proteins simultaneously. In addition to the library compounds cAMP was also injected at the beginning and end of each plate screening. Binding of cAMP to the C-linker/CNB of hHCN4 channels was used as a positive control for the screen. The pdb IDs of the PAS and C-linker/CNB(CNBH) domain structures are: 3OTF (HCN4 CNB), 4HOI (EAG PAS), 4F8A (EAG CNBH), 1BYW (ERG PAS), 2N7G (ERG CNBH), 4HP4 (ELK PAS) and 3UKN (ELK CNBH)

KCNH channels are assembled from four subunits with each subunit containing six transmembrane segments (TM1-TM6) (Fig. 1a) [1]. The voltage dependence of KCNH channels arises from the voltage-dependent conformational changes in the voltage sensors formed by the TM1-TM4 segments and the potassium selectivity is conferred by the selectivity filter formed by the TM5-TM6 loops from the four subunits [17–20]. While the transmembrane topology of KCNH channels is similar to other potassium channels, KCNH channels possess unique domains in their N and C-terminal intracellular regions. In the N-terminus KCNH channels contain Per-Arnt-Sim (PAS) and in the C-terminus cyclic nucleotide-binding homology (CNBH) domain [21, 22], connected to the TM6 segment with the C-linker. The PAS domain shares sequence and structural similarity with PAS domains from non-ion channel proteins, such as kinases and hydrolases [22, 23]. The CNBH domain shares sequence and structural similarity with the cyclic nucleotide binding (CNB) domains of the hyperpolarization-activated cyclic nucleotide-modulated (HCN) and cyclic nucleotide-gated (CNG) channels, and other CNB domain containing proteins, such as CAP and PKA [21, 24, 25]. Both PAS and CNB domains frequently function as ligand-binding domains in other proteins [23, 26–29]. Yet, there are no known small molecule binders for the PAS domains of KCNH channels. While CNB domains of HCN and CNG channels directly bind cyclic nucleotides, it was shown that cyclic nucleotides do not bind to the CNBH domains of EAG channels and bind with only low affinity to the CNBH domain of ERG channels [30]. So far only flavonoids were shown to directly bind to the CNBH domains of KCNH channels and affect their function [31, 32]. The presence of cavities suitable for small molecule binding in the PAS and CNBH domains of KCNH channels suggests that there is a potential for discovery of small molecule binders and necessitates the development of novel methods for their identification [33].

Here we describe a surface plasmon resonance (SPR)-based chemical library screening method for PAS and CNBH domains of KCNH channels to facilitate the discovery of small molecule binders of KCNH channels. One of the strength of the method is the ability to simultaneously screen for binding to the PAS and CNBH domains of all three KCNH channel subfamilies, which greatly simplifies the identification of sub-family specific small molecule binders. Using this method we identified undecylenic acid as an EAG PAS domain small molecule binder that binds to the PAS domain in a concentration-dependent and sub-family specific manner. Functional analysis of the effect of undecylenic acid on the currents from full-length EAG channels using inside-out patch-clamp recordings indicated that the compound inhibited currents from EAG channels in a concentration-dependent manner. Taken together, our results demonstrate the suitability of the SPR method for identifying small molecule binders of KCNH channels and identified undecylenic acid as an EAG PAS domain specific small molecule binder and EAG channel regulator. To our knowledge this is the first example of small molecule binder of the PAS domain of KCNH channels. Similar SPR-based approach should be applicable for small molecule binder discovery for other ion channels with ligand binding domains.

## Methods

### Protein expression and purification

PAS and C-linker/CNBH domains of mouse EAG (mEAG), human ERG (hERG) and human ELK (hELK), and the C-linker/CNB domain of human HCN4 (hHCN4) were subcloned into pETM11 bacterial expression vector containing an N-terminal 6-His affinity tag. The start and end of the amino acid sequence of the domains used in this study and their gene identifier (GI) numbers are indicated in Table 1. The proteins were expressed in BL21 (DE3) cells, and purified with Ni^2+^-NTA and size exclusion chromatography, as previously described [24, 30, 34]. The molecular weight of the purified proteins was verified with Coomassie Blue-stained gels and with mass spectrometry (electrospray) at Georgetown Proteomics and Metabolomics Core Facility. The purified proteins were stored at − 80 °C in small aliquots before use.

**Table 1:** Start and end amino acids and gene identifier (GI) numbers of the PAS, CNBH and CNB domains used in the study

	PAS	CNBH/CNB	GI number
mEAG1	7–136	552–708	26006801
hERG1	2–134	734–867	7531135
hELK1	5–136	536–678	26006815
hHCN4	N/A	521–723	Q9Y3Q4

### Protein immobilization for SPR

The purified domains of KCNH and HCN channels were immobilized on a NTA chip (GE Healthcare), as previously described [34]. Immobilizations of the proteins were performed in HBS-P buffer (150 mM NaCl, 10 mM HEPES, 0.05% (v/v) surfactant P20, pH 7.4). First the NTA sensor surface was activated with a 1 min injection of 0.5 mM NiCl_2_. The coupling of the Ni^2+^-NTA chip surface groups with the 6-His-tagged proteins was then achieved by 2.5 min injections of the proteins at ~ 200 nM concentrations for high density (HD) and ~ 10 nM for low density (LD) immobilization on the chip surface. After the initial capturing, the proteins were covalently cross-linked via 20 s injections of NHS-EDC carboxyl-reactive cross-linkers to prevent protein loss from the chip surface with successive analyte and buffer injections. This was followed by 20 s injection of 1 M ethanolamine to block the remaining reactive sites. The proteins were captured at ~ 1000–3000 RU (1 RU = 1 pg of protein per mm^2^).

### Spectrum collection library screening and application of individual compounds

SPR experiments were performed on a Biacore 4000 Instrument (GE Healthcare). The SPR response was reported in the resonance units (RU). 1 RU corresponds to 0.0001 degree of change in the reflected light angle. The SPR data were doubly corrected for the non-specific binding by subtracting the SPR response to the blank (buffer only) injections and also response to the control surface with no immobilized protein.

Spectrum Collection library screening experiments were performed at 25 °C in the running buffer B (150 mM KCl, 1 mM TCEP, 30 mM HEPES, pH 7.5), supplemented with 1% (v/v) DMSO. For the screening the library compounds supplied at 10 mM concentration in DMSO were diluted to 50 μM concentration in buffer B supplemented with 0.5% (v/v) DMSO to the final DMSO content of 1% (v/v) to precisely match the running buffer. The library compounds were injected over the chip surface for 60 s at a flow rate of 30 μl/min, followed by buffer only injections for 300 s.

For the dose-response experiments undecylenic acid, Spectrum Collection library compound identified as a small molecule binder of EAG channels, was injected over the range of concentrations in triplicates. To determine the binding affinity (K_d_), the steady state SPR responses (R_eq_) at 55 s after the start of the injection were plotted against the compound concentration and fitted with a Hill equation with Hill coefficient of 1, as described previously [32, 34]. The data analysis and fitting of the plots was performed in Origin (Microcal Software, Inc). For dose response experiments the error bars indicate the SEM. Omission of error bars indicates that the SEM was less than the size of the symbol in the figures.

### Spectrum collection chemical library and individual reagents

Spectrum Collection library of chemically active compounds (2560 small molecules in total) was purchased from MicroSource Discovery Systems Inc. The chemical compounds in the library included a combination of natural products from plants, animals and microorganisms, as well as drugs that have reached clinical trials and/or are currently marketed in the US and Europe. The library compounds were provided in thirty two 96-well plates at 10 mM concentration in DMSO. The first and last column of the 96-well plates were left empty by the supplier so that a control compounds can be included in the screen. We have shown that cAMP binding to the C-linker/CNB domain of HCN4 channels can be detected with SPR [34]. Therefore, 10 mM cAMP in DMSO was added into the first (A1) and last (H12) wells of the 96-well plates and used as a positive control for the screen.

For the SPR-based screening the compounds were first diluted to 50 μM concentrations in buffer B supplemented with 0.5% DMSO. This was achieved by transferring the compounds into 96-well plates (in the same orientation) with the appropriate volume of buffer for the dilution to 50 μM concentration. Then the library compounds at 50 μM concentration were transferred from the 96-well plates into 384-well plates using the placement described in the Additional file 1: Figure S1. E.g., after this transfer the solution in well A1 of the 96-well plate will be found in wells A1, C1, E1 and G1 of the 384-well plate. As a result, an initial 96-well plate of the Spectrum Collection library was represented in quadruplicates in one 384-well plate used for the SPR screen. This final transfer insured that the four flow cells of the Biacore 4000 will be simultaneously injecting the same library compound (e.g. in wells A1, C1, E1 and G1) over the seven PAS and CNB/CNBH domains of KCNH and HCN channels immobilized on the NTA sensor chip surface.

For the SPR-based screening experiments cAMP was purchased from Sigma. For the SPR dose-response and electrophysiology experiments undecylenic acid was purchased from Acros Organics.

### Electrophysiology

The cDNA encoding mEAG1 channels in pGH19 vector was kindly provided by G. Roberston (University of Wisconsin-Madison, Madison, WI). The cRNA was transcribed using the T7 mMessage mMachine kit (Thermo Fisher Scientific). Defolliculated *Xenopus laevis* oocytes were purchased from Ecocyte Bioscience (Austin, TX) and injected with the cRNA using a Nanoinject II oocyte injector (Drummond). Following a manual removal of the vitelline membrane oocytes were transferred to a handmade chamber containing bath solution for current recording. Currents were recorded at room temperature in the inside-out patch configuration [35] with Axopatch 200A patch-clamp amplifier (Molecular Devices) and pClamp10 software (Molecular Devices). The signals were digitized using Digidata 1550 (Molecular Devices).

Patch pipettes were pulled from borosilicate glass and had resistances of 0.50–1.2 MΩ after fire polishing. The intracellular (bath) and extracellular (pipette) solutions contained 130 mM KCl, 10 mM HEPES, 0.2 mM EDTA, pH 7.2. The mEAG1 currents were elicited by applying a series of 0.1 s voltage pulses (ranging from − 100 to + 70 mV in 10 mV increments) from a holding potential of − 80 mV, followed by a 0.15 s voltage pulse to − 100 mV. Currents were not leak subtracted.

To obtain conductance versus voltage curves for all electrophysiology experiments, peak tail current amplitudes were normalized to the largest peak tail current amplitude. These normalized data were then plotted against the test voltage, and were fit with a Boltzmann equation:

1 $$ \frac{G}{G_{\mathrm{max}}}=\frac{1}{1+{e}^{\left(\frac{V-{V}_{1/2}}{s}\right)}} $$

Where V represents the test voltage (mV), *V*_*1/2*_ is the midpoint activation voltage (mV), and *s* is the slope of the relation (mV). Deactivation (tail) currents were fit with a single-exponential equation.

For electrophysiology experiments 10 mM undecylenic acid stock in DMSO was diluted to 50 μM concentration in the bath solution with the final DMSO content of 0.5% (v/v). The subsequent dilutions for the dose-response experiments were prepared in the bath solution. In the inside-out configuration used in our experiments, the intracellular side of EAG channels containing the PAS domains was directly facing the bath solution. The bath solution was changed using a gravity-fed solution changer. To determine the IC50, the concentration of the compound at half-maximal current inhibition, the plots of the current inhibition versus the concentration of the small molecule binders were fitted with the Hill equation.

The data analysis and fitting of the plots were performed in Clampfit (Molecular Devices) and Origin (Microcal Software, Inc). The error bars on the figures correspond to the SEM. Statistical analysis was performed using Student’s t-tests. *P* values < 0.05 were considered significant. n represents the number of recordings.

## Results

### General description of the SPR-based screening method

To efficiently screen a library of chemical compounds for binding to the intracellular PAS and C-linker/CNBH domains of KCNH channels we developed a SPR-based screening method. The major steps of the method are summarized in Fig. 1. First, the isolated PAS and CNBH domains of KCNH channels were purified with the Ni^2+^-NTA affinity and size exclusion chromatography (Fig. 1a). We have shown that cAMP binding affinity to the C-linker/CNB domains of HCN4 channels can be reliably and accurately determined with SPR [34]. Therefore, C-linker/CNB domains of HCN4 channels were also purified and cAMP binding to these domains was used as a positive control for binding detection throughout the screen. The structures of the isolated PAS and C-linker/CNB(CNBH) domains of KCNH and HCN4 channels had been solved [22, 24, 25, 36–39], indicating that they are soluble and fold properly in solution. Structural comparison of the isolated domains with the recent cryo-electron microscopy structures of the full-length EAG, ERG and HCN1 channels indicates that the fold of the PAS and C-linker/CNB(CNBH) domains is essentially the same in the isolated form and as part of the full-length channel structures [34, 40–43]. Therefore, the small molecule binders of the isolated domains should also bind to the full-length channels.

The purified PAS and C-linker/CNB(CNBH) domains (seven proteins in total) were immobilized on the NTA sensor chip (Fig. 1b). The NTA chip contained 20 spots for protein immobilization and 4 flow cells for injecting solutions with library compounds over the immobilized proteins, with five spots (S1-S5) in each of the flow cells (FC1-FC4). For each FC, S3 was used as a control spot for non-specific binding. This spot was activated and blocked using similar coupling chemistry as other spots on the chip but with no immobilized protein. SPR response for S3 was subtracted from the SPR responses for other spots in the flow cell to account for the non-specific binding. For each FC, S1 and S2 had the same domains immobilized at high density (HD) for S1 and low density (LD) for S2, except for FC1, which had no protein immobilized on S1 and S2. Similarly, S4 and S5 had the same domains immobilized at LD density for S4 and HD for S5.

A library compound binding to the immobilized PAS or C-linker/CNB(CNBH) domains would be expected to change the total mass on the chip surface, which will be detected as a change in the SPR response. The SPR response quantifies the change in the angle of the reflected light in RU. To detect the binding of the library compounds to the immobilized domains, the SPR response was monitored continuously during the experiment. We used Spectrum Collection library of chemical compounds for the SPR screening. The Spectrum Collection library compounds, initially provided in thirty two 96-well plates, were distributed into thirty two 384-well plates as described in the methods and injected over the immobilized PAS and C-linker/CNB(CNBH) domains at 50 μM concentration (Fig. 1c). The four flow cells of Biacore 4000 were used to simultaneously inject the same library compound to all seven immobilized domains. This allowed a direct comparison of different library compound binding to the same immobilized domains and also of the same compound binding to the different PAS and C-linker/CNB(CNBH) domains.

Experiments on each 384-well plate were beginning and ending with cAMP injections over the immobilized proteins. The comparison of the SPR response for cAMP binding to the C-linker/CNB domain of HCN4 channels at the beginning and end of a plate screening was used as a measure of the stability of the immobilized C-linker/CNB(CNBH) domains on the chip surface. Only screening results for plates with similar cAMP binding response to the C-linker/CNB domain of HCN4 channels at the beginning and end of the plate screening were analyzed further while screening of the plates with different cAMP response was repeated.

With Biacore 4000 we were able to screen 3 to 4 384-well plates in 2 days while still detecting strong response for cAMP. This amounts to testing ~ 3000 binding events in 24 h, taking into account the injection of the compounds to 7 different proteins, each immobilized at low and high density. The data collected at the end of the screen was then analyzed with Microsoft Excel to identify small molecule binders of KCNH channels.

### Data analysis for the SPR-based screening method

To rank the library compounds in the order of the descending binding response to the immobilized protein the raw data were first corrected for the non-specific binding by subtracting the response to the buffer only injection and response to the control spot on the chip surface for the corresponding FC. The corrected data with negative values were then excluded from further analysis. Next was the comparison of the SPR responses for the LD and HD spots. If the library compound binding is specific the binding response for HD spots with the higher density of the immobilized domains should be larger than for the LD spots with the low density of the immobilized protein. Therefore, library compounds that did not follow this dependence were excluded from further analysis as non-specific binders.

**Fig. 2:**
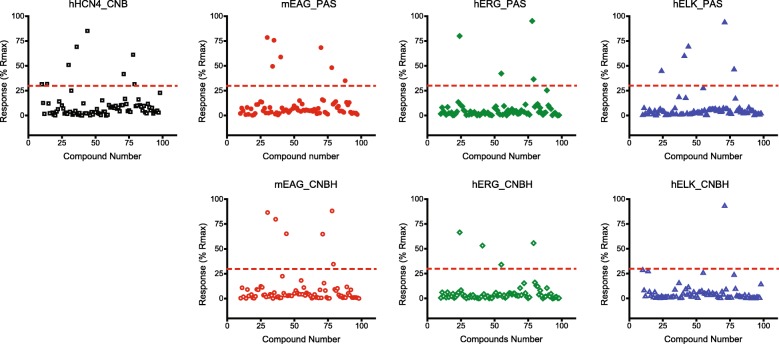
SPR screen results for a 96-well Spectrum Collection chemical library plate. Plots of the SPR responses in % of the maximal expected response (% R_max_) versus the Spectrum Collection library compound number in the 96-well plate for the indicated PAS and C-linker/CNB(CNBH) domains of KCNH and HCN channels. Library compounds with the % R_max_ < 30 were excluded from further analysis. The compounds were applied at 50 μM concentration

The SPR response is proportional to the change in the mass at the chip surface. Therefore, binding of a library compound with a smaller molecular weight would elicit smaller SPR response than binding of a heavier compound [44]. This difference is reflected in the maximum binding capacity (R_max_) for different compounds. The theoretical R_max_ can be calculated using the equation R_max_ = (MW_C_/MW_P_) × R × V, where MW_C_ is the molecular weight of the library compound, MW_P_ is the molecular weight of the immobilized protein, R is the immobilized protein level in RU measured as the protein gets captured on the surface, and V, is the valency of the immobilized protein/ library compound proposed interaction stoichiometry, which was set to 1 for our study, as we used low concentrations of the protein for immobilization [44]. To account for the differences in the molecular weight of the library compounds, % Rmax was calculated for each library compound. Representative SPR screen results plotted as % R_max_ for 96 compounds (cAMP + Spectrum Collection library compounds) distributed in quadruplicates on one 384-well plate are shown in Fig. 2.

The SPR response was sorted in the order of descending % R_max_ and library compounds with R_max_ > 30% were selected for further analysis. The cut off of 30% was chosen because most of the Spectrum Collection library compounds elicited responses with % R_max_ < 30% and yet sufficient number of ‘hit’ compounds was identified (Fig. 2). The cut off is arbitrary and can be adjusted if too many or too few hits are identified at the end of the screen. In addition to this cut off we used two other selection criteria to narrow down the number of compounds suitable for subsequent studies, as described below.

The next selection criteria were the structural and subfamily-dependent binding specificity of the library compounds. The PAS and C-linker/CNB(CNBH) domains have different structural fold. Therefore, if a library compound elicited similar binding response to both PAS and C-linker/CNB(CNBH) domains it was excluded from further analysis as a non-specific binder. Finally, the SPR binding responses were analyzed for subfamily-specificity. The library compounds that exhibited binding response to domains from one subfamily of KCNH but no binding or substantially lower binding response to the other KCNH subfamilies were ranked in the order of descending response and selected for more in-depth analysis.

Out of 2560 Spectrum Collection library compounds 59 were identified as subfamily- and/or structure-specific small molecule binders when injected at 50 μM concentration to the PAS and C-linker/CNBH domains of KCNH channels. This amounts to the hit rate of ~ 0.4% for each of the six different PAS and C-linker/CNBH domains used in the screen.

### Undecylenic acid binds to the PAS domain of mEAG channels in a concentration-dependent manner with a sub-family and structural specificity

**Fig. 3:**
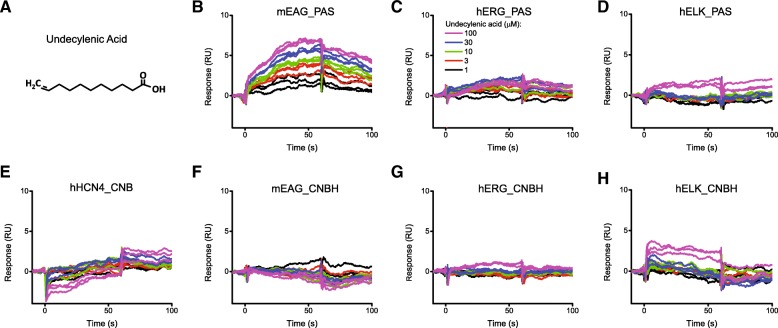
Undecylenic acid is a novel small molecule binder of the PAS domain of mEAG channels. **a** Chemical structure of undecylenic acid identified as the mEAG PAS domain specific binder with the SPR-based screening method. SPR sensorgrams recorded for the immobilized PAS domains of mEAG (**b**), hERG (**c**) and hELK (**d**), and C-linker/CNB(CNBH) domains of hHCN4 (**e**), mEAG (**f**), hERG (**g**) and hELK (**h**) channels recorded with the indicated concentration of undecylenic acid

To further validate the functional significance of the screen we investigated an effect of undecylenic acid, one of the mEAG_PAS domain small molecule binders identified by our screen. We determined the SPR response for undecylenic acid over the range of concentrations for all seven target proteins. Application of undecylenic acid (structure shown in Fig. 3a) to the PAS domain of mEAG channels increased the SPR response in a concentration-dependent manner (Fig. 3b). Consistent with the sub-family specific binding of undecylenic acid observed in the initial SPR-based screen, no substantial binding to the PAS domains of hERG and hELK channels was detected over the examined concentration range (Fig. 3c and d). Consistent with the structural specificity observed during the initial SPR screen, no substantial binding was detected to the C-linker/CNB(CNBH) domains of hHCN4, mEAG and hERG channels over the examined concentration range (Figs. 3e, f and g). At 100 μM concentration undecylenic acid showed some binding to the C-linker/CNBH domain of hELK channels (Fig. 3h), however, the SPR response was much smaller than the SPR response for the PAS domain of EAG channels at the same concentration of the compound.

**Fig. 4:**
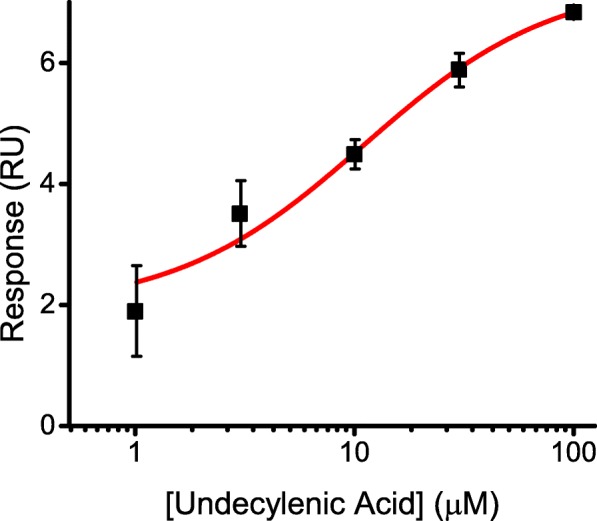
Determination of the binding affinity of undecylenic acid for the mEAG PAS domain. Plots of the SPR response at 55 s after the start of the injection versus total undecylenic acid concentration for sensorgrams shown in Fig. 3b. The lines represents fit of the data with Hill equation. The binding affinity for undecylenic acid was ~ 11 μM

To determine the binding affinity of undecylenic acid the SPR responses reported in Fig. 3b were plotted against the small molecule concentrations (Fig. 4). Fitting of the dose-response plot for undecylenic acid revealed the binding affinity of ~ 11 μM. In summary, the SPR dose response experiments confirmed the sub-family and structural specificity of undecylenic acid binding to the PAS domains of mEAG channels revealed by the initial SPR screen.

### Undecylenic acid inhibits currents from mEAG channels

**Fig. 5:**
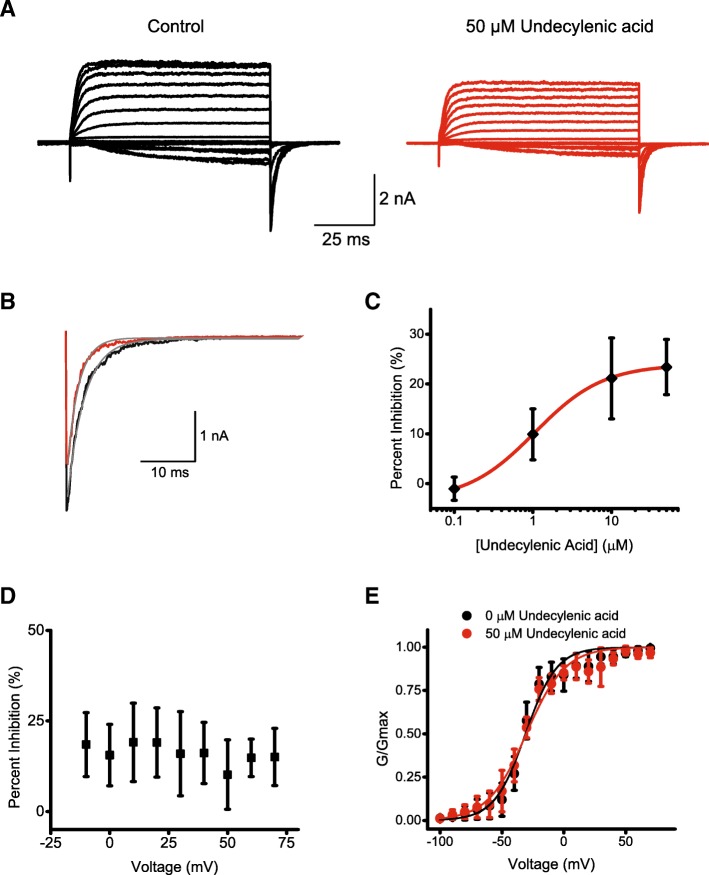
Undecylenic acid inhibits currents from mEAG channels. **a** Representative mEAG current traces recorded in the inside-out configuration in the absence (black) and presence (red) of 50 μM undecylenic acid. **b** The tail current recorded at − 100 mV after a voltage step to + 70 mV in the absence (black) and presence (red) of 50 μM undecylenic acid. The grey lines represent fits of the tail currents with a single exponential function with the time constants of deactivation of 3.1 ms in the absence and 2.1 ms in the presence of undecylenic acid. **c** Plots of the percent inhibition of tail currents versus the undecylenic acid concentration. Tail currents were recorded at − 100 mV after a voltage step to 70 mV. The line represents fit of the data with Hill equation with the IC50 of 1.06 ± 0.03 μM. **d** Plot of the averaged percent inhibition of tail currents versus voltage obtained in the presence of 50 μM undecylenic acid (*n* = 4). **e** Conductance-voltage relationship in the absence (black) and presence (red) of 50 μM undecylenic acid. The black and red lines represent fits of the conductance-voltage relationships in the absence (black) and presence (red) of 50 μM undecylenic acid with a Boltzmann function with V_1/2_ of − 30.9 ± 1.5 mV and − 30.3 ± 1.6 mV, respectively

To determine the functional effect of undecylenic acid mEAG channel currents were recorded in the absence and presence of 50 μM of the compound (Fig. 5a). Undecylenic acid decreased currents from mEAG channels recorded at + 70 mV by 19.9% and tail currents by 25.8% (Fig. 5a and b). Similar effect was observed in n of 4 experiments performed on membrane patches from different oocytes.

Undecylenic acid inhibited mEAG channel currents in a concentration-dependent manner with IC50 of 1.1 ± 0.02 μM (Fig. 5c, *n* = 4). The decrease in the current was voltage-independent and did not change the deactivation time constant (τ) or shift the half-maximal activation voltage (V_1/2_) (Fig. 5d and e). The average V_1/2_ was 30.9 ± 1.4 mV in the absence and − 30.3 ± 1.5 mV in the presence of undecylenic acid. The average τ was 2.4 ± 0.7 ms in the absence and 2.0 ± 0.7 ms in the presence of undecylenic acid (n = 4, *P* = 0.46, *t* test). The intracellular solution with 50 μM undecylenic acid contained 0.5% DMSO. Application of 0.5% DMSO alone had no effect on mEAG channel currents (n = 4, Additional file 1: Figure S2).

## Discussion

To identify small molecule binders of intracellular PAS and C-linker/CNBH domains of KCNH channels we developed SPR-based chemical library screening method. This method should be applicable to other ion channels containing ligand-binding domains and should facilitate the discovery of novel ion channel modulators. Using the SPR-based method, we screened Spectrum Collection chemical library and identified a pool of library compounds that bind to the PAS or C-linker/CNBH domains of KCNH channels in a subfamily-specific and/or structure-specific manner. To test the functional significance of the screen, we investigated the binding and functional effect on EAG channel currents of undecylenic acid, one of the identified library compounds that binds to the PAS domain of mEAG channels. SPR response recorded over the rage of undecylenic acid concentrations indicated that the compound binds to the PAS domain of EAG channels in a concentration-dependent manner while showing substantially weaker binding to the PAS domains of ERG and ELK channels, and C-linker/CNB(CNBH) domains of HCN and KCNH channels. Functional examination of the effects of undecylenic acid on the currents from mEAG channels indicated that the compound inhibited mEAG channel currents in a concentration-dependent manner. Taken together, our results indicate that the SPR-based chemical library screening method is well suited for identifying subfamily specific KCNH channel small molecule binders.

The major advantage of the SPR-based screening method is the capability of simultaneous screening of several target proteins for binding of the library compounds. This is especially relevant to a family of homologous proteins with sufficient diversity to support binding of distinct small molecule binders. For the KCNH channels this amounted to the opportunity to test ligand binding in the same experiment for the PAS and C-linker/CNBH domains of all three KCNH subfamilies, and C-linker/CNBD of HCN4 channels using Biacore 4000 SPR instrument (Figs.1 and 2). The distinct structural fold of the PAS and CNB/CNBH domains provided further means to select for structural specificity in the binding among the screened library compounds. The PAS domains of KCNH channels share 54 to 75% identity in their amino acid sequence, and the CNBH domains of KCNH channels share 62 to 65% amino acid identity (Additional file 1: Table S1). This difference in the amino acid sequence was enough to support mEAG PAS domain specific binding of undecylenic acid (Fig. 3).

Undecylenic acid is an unsaturated fatty acid used for the treatment of nail fungal infections [45, 46]. Our results indicate that undecylenic acid elicited concentration-dependent increase in the SPR response upon injection to the immobilized PAS domain of EAG channels while showing no concentration-dependent changes upon injection to the immobilized PAS domains of ERG and ELK channels (Fig. 3). The binding was structure specific as only a very weak binding was observed to structurally dissimilar CNB/CNBH domains of KCNH and HCN channels. Moreover, undecylenic acid decreased currents from mEAG channels recorded in the inside-out configuration of the patch-clamp technique in a concentration-dependent manner (Fig. 5). To our knowledge, undecylenic acid is the first identified regulator of KCNH channels that binds to the PAS domain of these channels.

The SPR-based method described here has several other advantages for novel ion channel ligand identification. There is no need for the development of a radioactive or fluorescently labeled ligand for the SPR-based screening method. This enables ligand identification for orphan receptors with no known ligands and does not restrict the ligand identification to the ligands that only bind to the known or predicted binding pockets. The SPR method detects direct binding of the ligands to the immobilized protein, simplifying the interpretation of the mechanism in comparison with many cell-based assays. In addition, the SPR-based screening method requires very little amounts of protein for screening. The stringency of the screen could be adjusted by changing the % cut off and the concentration of the applied compounds. The ranking of the compounds according to their SPR response, and structural and sub-family specificity should provide further means to prioritize the future studies of the individual hit compounds.

## Conclusion

Here we described an SPR-based chemical library screening method for KCNH channels that allows identification of subfamily- and structure-specific small molecule binders. Using this method we screened the Spectrum Collection chemical library and identified undecylenic acid as the mEAG PAS domain specific small molecule binder and EAG channel inhibitor. The SPR-based chemical library screening method should be applicable to other target proteins and should enable identification of the target-specific novel small molecule binders in the future.

## Additional file

Additional file 1: **Table S1.** PAS and CNBH domain amino acid identity (%) for KCNH channels. **Figure S1.** Placement map for transferring 96-well plates with Spectrum Collection library compounds into 384-well plates for the SPR-based screening. The figure illustrates a quadruplicate representation of a 96-well plate of the Spectrum library in a 384-well plate used for the SPR screening. Rows and columns for the 384-well plate are indicated in red and for the 96-well plate in black. A1 well of the 96-well plate is colored in blue to illustrate the quadruplicate representation. **Figure S2.** (A) Representative mEAG current traces recorded in the inside-out configuration in the absence (black) and presence (gray) of 0.5% DMSO. (B) The tail current recorded at − 100 mV after a voltage step to 70 mV in the absence (black) and presence (gray) of 0.5% DMSO. (PDF 886 kb)

## Data Availability

The information on the chemicals used in this manuscript could be requested from the corresponding author.

## Abbreviations

CNB: Cyclic nucleotide-binding
CNBH: Cyclic nucleotide-binding homology
DMSO: Dimethyl sulfoxide
EAG: Ether-à-go-go
ELK: EAG-like K+
ERG: EAG-related gene
PAS: Per-Arn-Sim
SPR: Surface plasmon resonance
