# Supplementary material for: Identification of undecylenic acid as EAG channel inhibitor using surface plasmon resonance-based screen of KCNH channels
Source: BMC Pharmacol Toxicol. 2019 Jul 17;20:42. doi: 10.1186/s40360-019-0324-8 (PMC6637479; doi:10.1186/s40360-019-0324-8)
Supplement: Supplementary file 1 — Table S1. PAS and CNBH domain amino acid identity (%) for KCNH channels. Figure S1. Placement map for transferring 96-well plates with Spectrum Collection library compounds into 384-well plates for the SPR-based screening. The figure illustrates a quadruplicate representation of a 96-well plate of the Spectrum library in a 384-well plate used for the SPR screening. Rows and columns for the 384-well plate are indicated in red and for the 96-well plate in black. A1 well of the 96-well plate is colored in blue to illustrate the quadruplicate representation. Figure S2. (A) Representative mEAG current traces recorded in the inside-out configuration in the absence (black) and presence (gray) of 0.5% DMSO. (B) The tail current recorded at − 100 mV after a voltage step to 70 mV in the absence (black) and presence (gray) of 0.5% DMSO. (PDF 886 kb) [file 40360_2019_324_MOESM1_ESM.pdf]

Supporting Table II

PAS and CNBH domain amino acid identity (%) for KCNH channels

PAS	mEAG	hERG	hELK
mEAG		54	65
hERG			75

CNBH	mEAG	hERG	hELK
mEAG		65	62
hERG			62

	1	2	3	4	5	6	7	8	9	10	11	12	13	14	15	16	17	18	19	20	21	22	23	24
A	A1	B1	A2	B2	A3	B3	A4	B4	A5	B5	A6	B6	A7	B7	A8	B8	A9	B9	A10	B10	A11	B11	A12	B12
B	C1	D1	C2	D2	C3	D3	C4	D4	C5	D5	C6	D6	C7	D7	C8	D8	C9	D9	C10	D10	C11	D11	C12	D12
C	A1	B1	A2	B2	A3	B3	A4	B4	A5	B5	A6	B6	A7	B7	A8	B8	A9	B9	A10	B10	A11	B11	A12	B12
D	C1	D1	C2	D2	C3	D3	C4	D4	C5	D5	C6	D6	C7	D7	C8	D8	C9	D9	C10	D10	C11	D11	C12	D12
E	A1	B1	A2	B2	A3	B3	A4	B4	A5	B5	A6	B6	A7	B7	A8	B8	A9	B9	A10	B10	A11	B11	A12	B12
F	C1	D1	C2	D2	C3	D3	C4	D4	C5	D5	C6	D6	C7	D7	C8	D8	C9	D9	C10	D10	C11	D11	C12	D12
G	A1	B1	A2	B2	A3	B3	A4	B4	A5	B5	A6	B6	A7	B7	A8	B8	A9	B9	A10	B10	A11	B11	A12	B12
H	C1	D1	C2	D2	C3	D3	C4	D4	C5	D5	C6	D6	C7	D7	C8	D8	C9	D9	C10	D10	C11	D11	C12	D12
I	E1	F1	E2	F2	E3	F3	E4	F4	E5	F5	E6	F6	E7	F7	E8	F8	E9	F9	E10	F10	E11	F11	E12	F12
J	G1	H1	G2	H2	G3	H3	G4	H4	G5	H5	G6	H6	G7	H7	G8	H8	G9	H9	G10	H10	G11	H11	G12	H12
K	E1	F1	E2	F2	E3	F3	E4	F4	E5	F5	E6	F6	E7	F7	E8	F8	E9	F9	E10	F10	E11	F11	E12	F12
L	G1	H1	G2	H2	G3	H3	G4	H4	G5	H5	G6	H6	G7	H7	G8	H8	G9	H9	G10	H10	G11	H11	G12	H12
M	E1	F1	E2	F2	E3	F3	E4	F4	E5	F5	E6	F6	E7	F7	E8	F8	E9	F9	E10	F10	E11	F11	E12	F12
N	G1	H1	G2	H2	G3	H3	G4	H4	G5	H5	G6	H6	G7	H7	G8	H8	G9	H9	G10	H10	G11	H11	G12	H12
O	E1	F1	E2	F2	E3	F3	E4	F4	E5	F5	E6	F6	E7	F7	E8	F8	E9	F9	E10	F10	E11	F11	E12	F12
P	G1	H1	G2	H2	G3	H3	G4	H4	G5	H5	G6	H6	G7	H7	G8	H8	G9	H9	G10	H10	G11	H11	G12	H12

Supplemental Figure 1. Placement map for transferring 96-well plates with Spectrum Collection library compounds into 384-well plates for the SPR-based screening. The figure illustrates a quadruplicate representation of a 96-well plate of the Spectrum library in a 384-well plate used for the SPR screening. Rows and columns for the 384-well plate are indicated in red and for the 96-well plate in black. A1 well of the 96-well plate is colored in blue to illustrate the quadruplicate representation.

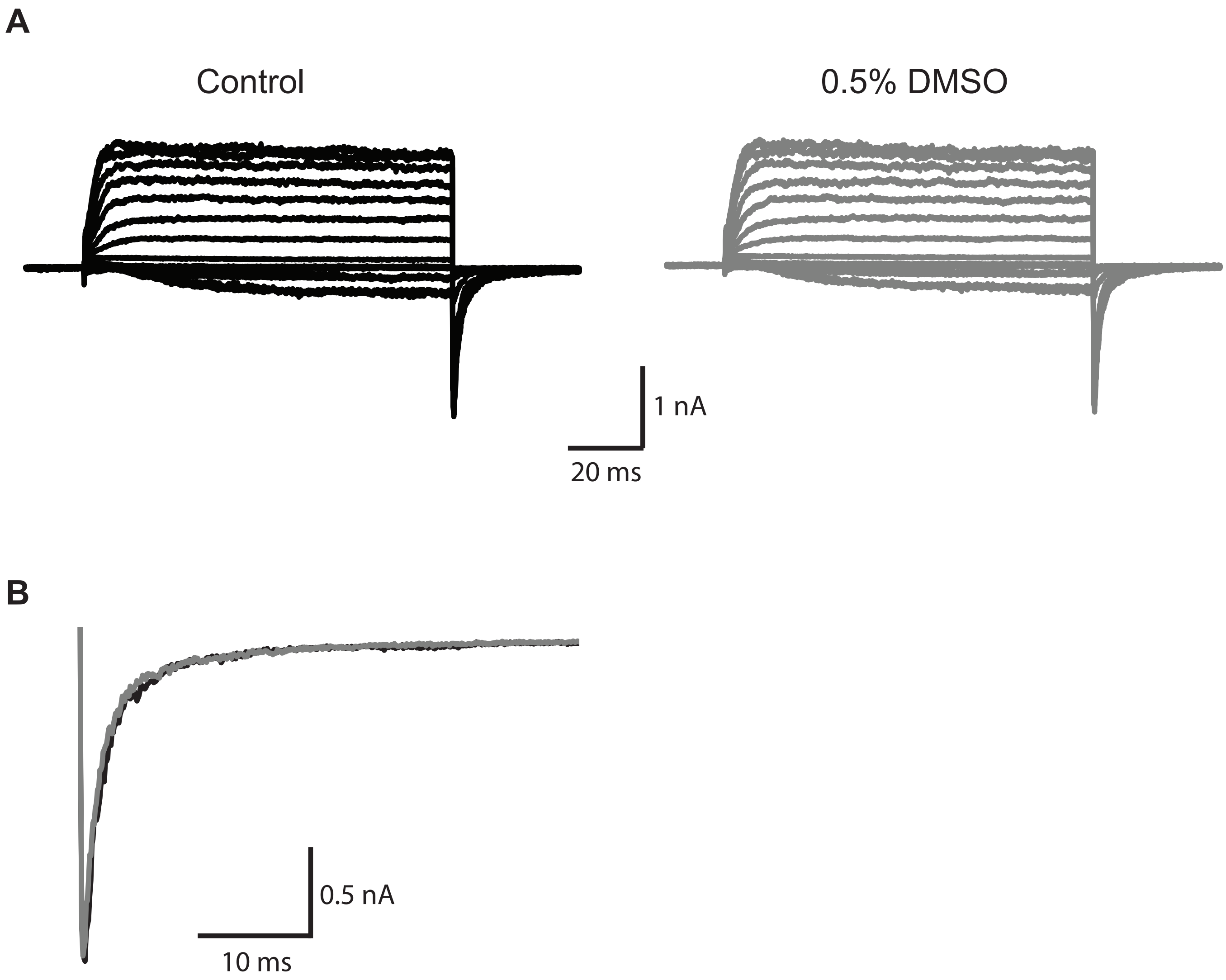

Supplimental Figure 2. (A) Representative mEAG current traces recorded in the inside-out configuration in the absence (black) and presence (gray) of 0.5% DMSO. (B) The tail current recorded at – 100 mV after a voltage step to 70 mV in the absence (black) and presence (gray) of 0.5% DMSO.
